# Anthelmintic Effect of Chitosan‐Encapsulated Bromelain on Gastrointestinal Nematodes in Naturally Infected Goats in Kenya

**DOI:** 10.1155/japr/3931872

**Published:** 2026-01-05

**Authors:** Ahmota Romain Daïba, Issa Youssouf Adoum, Maina Ngotho, John Maina Kagira, Naomi Maina

**Affiliations:** ^1^ Department of Livestock Science and Technology, National Higher Institute of Science and Techniques of Abeche, Abéché, Chad; ^2^ Department of Biology, Higher Normal School of N′Djamena, Abéché, Chad; ^3^ Department of Clinical Studies, University of Nairobi, Nairobi, Kenya, uonbi.ac.ke; ^4^ Department of Animal Sciences, Jomo Kenyatta University of Agriculture and Technology, Nairobi, Kenya, jkuat.ac.ke

**Keywords:** anthelmintic efficacy, bromelain, chitosan, encapsulation, goat

## Abstract

The emergence of anthelmintic resistance calls for the development of alternatives, including medicinal plant extracts. The present study was aimed at assessing the efficacy of chitosan‐encapsulated bromelain (EB) as an oral solution drug against GIN in goats. Standard methods were followed to extract bromelain from the pineapple peels and the conventional manufacturing procedure used to make an oral solution of EB. The *in vivo* study was performed on 20 healthy male goats that were naturally infected with GIN. The goats were randomly divided into four groups, each of which consisted of three treatment groups: 90 and 270 mg/kg EB, 7.5 mg/kg albendazole, and negative control. The oral solution of EB was administered orally once daily for a period of 3 days. The fecal egg counts (FECs) were undertaken using a McMaster technique. The goats were monitored for clinical signs on a daily basis, and their body weight was reported on a weekly basis. Weekly blood samples were collected and analyzed for the presence of packed cell volume (PCV), aspartate aminotransferases (ASTs), alanine aminotransferases (ALTs), urea, and creatinine. The goats were sacrificed and assessed for gross and histopathology analyses. The results showed that at 28 days′ posttreatment there was a significantly higher reduction of FEC of albendazole (98.58%) and 270 mg/kg (81.73%) groups than 90 mg/kg (59.84%). No clinical manifestations or mortality were observed in the goats during the monitoring period. All animals exhibited normal levels of PCV, AST, ALT, creatinine, and urea. The pathology findings also revealed no toxic effects on the goat organs. In conclusion, the oral solution of EB was effective in reducing the GIN burden and safe for use in goats. Further investigations are essential to establish better formulation and adjusting the dosage regimens to increase the efficacy on GIN.

## 1. Introduction

Goat breeding across the world is greatly impacted by gastrointestinal nematodes (GINs), which provide a serious health risk to the animals [1, 2]. These endoparasites have detrimental effects on animal production, including a decrease in milk, a loss of weight gain, and in extreme cases, death [3]. Herd mortality rates surpassing 40%, and weight losses of 6–12 kg/year per animal may occur [4]. The incidence of GIN is influenced by agroclimatic factors such as temperature, humidity, vegetation, and animal behavior [5]. However, *Haemonchus* spp., *Trichostrongylus* spp., *Oesophagostomum* spp., *Ostertagia* spp., *Nematodirus* spp., and *Cooperia* spp. are the most frequent GIN that impact goats [6, 7]. Among these species, *Haemonchus contortus* is considered the most dangerous due to its high incidence and pathogenicity infecting goats [8, 9].

Principally, the nematodes are controlled by use of synthetic medications on a global scale [10]. Even with frequent strategic management, treatments for nematodes can be costly and only partially successful for resource‐poor farmers [11]. In addition, repeated and inadequate use of these medications has resulted in a severe and alarming level of anthelmintic resistance among parasite populations [12, 13]. Also, the use of anthelmintic drugs has raised concerns among consumers regarding the presence of drug residues in animal products [14, 15]. The advent of anthelmintic medicine resistance increased the demand for alternative GIN controlling products. Recently, plant extracts such as cysteine proteinase plants have been investigated as environmentally sustainable alternatives for managing parasitoses and have been shown to be highly effective [16, 17]. One of the therapeutic plants used is pineapples (*Ananas comosus*) that found to have antiparasitic and antibacterial properties in animals [17–21]. Recent research has shown that the pineapple enzyme bromelain is 68% of effective against gastrointestinal worms in goats after administered 30 mg/kg for 14 days [20]. The main challenges of bromelain is to maintain stability within the gastrointestinal system of goats. In recent years, some studies have shown that the chitosan‐encapsulated bromelain (EB) can stabilize and maintain the activity of protein throughout the digestive system [19, 20]. Consequently, the objectives of this study are aimed at evaluating the anthelmintic efficacy of bromelain encapsulated in chitosan, as well as to assess the potential adverse effects of the medicine.

## 2. Material and Methods

### 2.1. Ethics Statement

The Animal Ethics Committee of the Department of Veterinary Physiology and Anatomy at the University of Nairobi provided the ethical authorization necessary for the approval of this protocol study (REF: FVMBAUEC/2020/339). The study conformed to the protocols, animal husbandry practices, and design that were approved by the Committee.

### 2.2. Experimental Site

The current study was performed from January to February 2022, at Jomo Kenyatta University of Agriculture and Technology (JKUAT) animal facility, located in Juja, in Kiambu County, Kenya. The University is located at longitude 37°00 E and latitude 1°05 S, and it lies at an altitude of 1525 m above sea level with rainfall bimodal ranging from 500 to 1300 mm while the average temperature is 19.5°C [22].

### 2.3. Extraction and Encapsulation of Bromelain in Chitosan

Bromelain was extracted from the skins of pineapples (*Ananas comosus*) which was obtained from Juja subcounty market in Kenya. The protein was isolated using the method described previously by [20]. In brief, 500 g of fresh peels of pineapples were blended in 1000 mL of sodium acetate buffer solution (pH 7.4) that was prepared by dissolving 8.2 g in 800 mL of distilled water. The filtrate was labeled as crude extract after the solution was filtered through a sieve (model V3CF‐250 *μ*m size). The crude extract that resulted was precipitated by incubating it at + 4°C overnight with 40% ammonium sulfate. Then, the extracted bromelain was purified using a 12‐kDa molecular weight cut‐off (MWCO) dialysis membrane after 24 h. The bromelain was encapsulated in chitosan (Sigma Aldrich, United States) using the ionic gelation procedure, as outlined by Hunduza et al. [19]. The pellet that was obtained after encapsulation was frozen at −20°C and subsequently −70°C (MDF‐U5312‐PE, Panasonic freezer). The freeze‐dryer (MRC, Model FDL‐10N‐50‐BA, Israel) was employed to dry the product. The powdered EB in chitosan was collected and stored at + 4°C. Bromelain′s successful encapsulation in chitosan nanoparticles was confirmed through Fourier transform infrared spectrophotometer (FTIR) analysis. After that, the protease activity was assessed using the casein enzymatic assay, as described by Wasso et al. [20].

### 2.4. Oral Solution of EB Formulation

EB oral solution was prepared using the procedure described previously [23, 24]. In the manufacturing process, the solvent contained in the excipients such as potassium sorbate (0.75 g)), sucrose (150 g), xanthan gum (1.5 g), Tween 20 (0.5 mL), and propylene glycol (100 mL) were combined for the preparation of 500 mL of an oral solution of EB with a concentration of 100 mg/mL [25]. Casein enzymatic assays were carried out to evaluate the proteolytic activity and specific activity at diverse storage conditions (at + 4°C and at room temperature, 25°C) [26].

### 2.5. Origin of Animals

Twenty (20) small East African goats were acquired from farmers in Makima Ward, Embu County, Kenya. Strongyle nematodes were naturally present in all goats, with a concentration of over 2000 ova per gram of excrement. The goats had an average age of 15 months and weighed between 16 and 29 kg. The animals were tagged with ear tags for simple identification during the 2‐week acclimatization period. The goats were housed in the JKUAT goat house and were provided with 1.5 kg of wheat hay thrice daily; 1 kg of concentrate consisting of beet liquid molasses, maize germ, and soybean meal (Aroma Feed Suppliers, Kenya); and feed blocks minerals (Aroma Feed Suppliers, Kenya).

### 2.6. Study Groups and Sampling of Animals

Fecal samples were collected from goat rectums using sterile gloves and examined using the McMaster procedure to determine the number of eggs per gram (EPG) of feces. All of the animals used in the studies had EPG results higher than 2000. The goats were divided randomly into four (4) treatment groups. Groups 1 and 2 were treated with 90 and 270 mg/kg of EB oral solution, respectively, while Group 3 served as the positive control, receiving 7.5 mg/kg of albendazole (Sigma‐Aldrich), and Group 4 was untreated goats (negative control). The levels presented were selected based on previous studies on the toxicity and efficacy of bromelain encapsulated in chitosan [20]. The medication was given orally every morning for three (3) days, and the goats were observed for 28 days after the final day of drug administration.

### 2.7. In Vivo Assessment of Anthelmintic Efficacy

Fecal egg count reduction (FECR) was used to assess anthelmintic efficacy of the oral solution of EB formulated. During the 4 weeks of the monitoring period, once weekly rectal feces samples were collected from goats. The feces were examined using a modified McMaster technique to measure the fecal egg counts (FECs) [27]. FECR percentage was calculated using the formula as previously described [27]: *%*FECR = 100 × [1 − (*T*2/*T*1)] with *T*1 is the mean pretreatment FEC and *T*2 is the mean posttreatment FEC in the treatment group (at Day 7, Day 14, Day 21, and Day 28 posttreatment).

### 2.8. Evaluation of Anthelmintic Efficacy of EB Oral Solution Against GIN Using Multiplex PCR (mPCR)

The mPCR was carried out to identify the species of nematodes present in gastrointestinal transit (GIT) before and after treatment. The efficacy was assessed at Day 7 and Day 28 posttreatment. DNA was extracted using *Quick-*DNA Tissue/Insect Miniprep Kit (Zymo Research Corp., Irvine, CA, United States) as described by the manufacturer [28]. After elution, DNA was stored at −20°C until used. Prior to evaluating EB anthelmintic efficacy, mPCR was optimized for detection of the main GIN strongyles prevalent in Kenya goats. A 5‐time Master Mix (Solis Biodyne) was employed. Specific primers for the nematode species (Table 1) were designed using Primer Blast tool in National Center for Biotechnology Information (NCBI) (https://www.ncbi.nlm.nih.gov/tools/primer-blast/), while reference sequences of the genes were retrieved from GenBank in FASTA format. Each pair of primers obtained considering GC contents and primer melting temperature (Tm) were tested in sequence manipulation suite (https://www.bioinformatics.org/sms2/pcr_primer_stats.html). The mPCR mixture included 2 *μ*L of DNA template, 1 *μ*L of forward and reverse primers, and 25 *μ*L of Firepol Master Mix. The reaction volume was increased to 50 *μ*L by adding DNA‐free water. The polymerase chain reaction was carried out under the following conditions: initial denaturation at 95°C for 4 min, followed by 35 cycles at 95°C for 30 s, 50°C for 45 s, 72°C for 2 min, and final extension at 72°C for 7 min. The amplicons were electrophoresed on a 2% TAE agarose gel (50 min , 70 V), stained with ethidium bromide, and the DNA migration and resolution pattern was evaluated.

**Table 1:** Sequences of genus‐specific primers of gastrointestinal nematode parasites of ruminants.

**Nematode species**	**Primer sequences in 5** ^′^ **-3** ^′^ **direction**	**Fragment size in bp**	**Tm (°C)**	**Accession number**
*Haemonchus contortus*	F: TTGAAGAGGGTTTGGTGTAA	320	50	NC‐010383.2
	R: TTCTGGCACAATATGAACTG			
Common ITS2	F: CACGAATTGCAGACGCTTAG	380	50	Bisset et al. [29]
	R: GCTAAATGATATGCTTAAGTTCAGC			
*Trichostrongylus vitrinus*	F: TTAGGTCATCCGGGTAGTAG	506	50	MK275237.1
	R: TAGGCACGAGAATCCAAATC			
*Trichostrongylus axei*	F: TTGTGGTCTGGGATAATTGG	552	50	KJ755059.1
	R: TTCTACCTGGGTGACCTAAA			
*Trichostrongylus colubriformis*	F: GATTGTTCATGCGAAGTTCC	418	50	AB908960.1
	R: GAGTTAGCCACACTGTAGAA			
*Nematodirus filicollis*	F: ATTTACCACGAATTGCAGAC	142	50	AF194140.1
	R: AAGTACCATTCGACACACAG			
*Oesophagostomum* spp	F: TTGTCACTGTTAGAGCGTTT	78	50	AJ889570.1
	R: GCAAATGACATGAAACCACA			
*Ostertagia ostertagi*	F: GCTGGTGCTATCACTATGTT	216	50	AB246113.1
	R: CACTATACCCAATGAACCGA			

### 2.9. Assessment of Acute Toxicity Effect

Following the administration of medication, the animals were examined for the first 30 min and subsequently at regular intervals throughout the first 24 h. Particular attention was provided during the first 4 h and then daily for the next 28 days to evaluate any changes in general behavior and other physiological activities [30, 31]. The rectal temperature of goats was taken every day in the morning (8 h30–9 h30 am) using a digital thermometer (Kruuse Digital Thermometer; Jorgen Kruuse). The body weight of the animals was measured weekly. Weekly, 2 mL of blood was collected from each animal′s jugular vein and placed in a 4‐mL EDTA blood collection container. The PCV was calculated using the micro‐hematocrit technique [32]. Blood samples were then centrifuged at 14,000 rpm for 10 min to collect plasma. The plasma samples were tested for aspartate aminotransferases (AST), alanine aminotransferases (ALT), urea, and creatinine levels using standard diagnostic test kits on an Automated Clinical Biochemistry Analyzer (Reflotron Plus System, model: Cobas 4800 Detection Analyzer; India) [20, 33].

The goats were sacrificed on the 28^th^ day, after the final day of medication administration, and gross pathology was done as previously reported [34]. Sections of the liver, kidney, spleen, and heart were taken and kept in 10% buffered formalin for 24 h before being processed for histology, as previously reported [35].

### 2.10. Statistical Analysis

The acquired data was typed into and analyzed using Graph Pad Prism 8.4.3 for statistical analysis. Before proceeding with any additional statistical tests, descriptive statistics (means and standard deviations) were generated. FECR, PCV, weight, temperature, and biochemical parameters were compared across groups using paired sample *t*‐tests, with *p* value < 0.05 indicating statistical significance.

## 3. Results

### 3.1. Bromelain Oral Solution pH and Activity

The EB oral solution was stored at different temperatures for 2 weeks. The protease activity of EB oral solution was 0.106 ± 0.01 and 0.084 ± 0.009 U/mL for drugs stored at + 4°C and room temperature (25°C), respectively. EB oral solution kept at + 4°C had significantly greater proteolytic activity (*p* < 0.005) than that stored at ambient temperature. The pH of the EB oral solution varied from 4.7 ± 0.3 to 4.6 ± 0.4. There were no significant differences (*p* > 0.05) between the pH of the EB oral solution kept at + 4°C and that kept at ambient temperature (25°C).

### 3.2. In Vivo Assessment of Anthelminthic Efficacy of EB Oral Solution

At Day 0, the average of EPG for the 270 mg/kg, 90 mg/kg EB oral solution, and positive control groups were 2375, 2300, and 2970, respectively. At Day 7 posttreatment, the FECR% for goats treated with albendazole, 270 and 90 mg/kg EB oral solution were 79.45%, 66.98% and 50.42%, respectively. On the 21st day of medical treatment, the FECR levels were 98.58%, 81.73%, and 59.84% for the albendazole, 270, and 90 mg/kg EB oral solution groups, respectively. While in negative control, FEC increased from 2350 eggs to 2749.20 ± 328.51 eggs (121.80*%* ± 17.41*%*). The goats treated with 270 mg/kg oral solution had a significantly higher decrease in GIN eggs (*p* < 0.05) compared to those treated with 90 mg/kg. Table 2 shows a substantial (*p* < 0.05) difference in FECR% between goats treated with albendazole and those treated with 270 mg/kg EB oral solution.

**Table 2:** Mean (%) of fecal egg count reduction of goats treated with EB and albendazole.

**Treatment group**	**Day 7**	**Day 14**	**Day 21**	**Day 28**
270 mg/kg EB	66.98 ± 6.32^a^	78.59 ± 5.58^a^	81.73 ± 5.01^a^	76.01 ± 7.62^a^
90 mg/kg EB	49.60 ± 7.13^b^	60.38 ± 5.81^b^	59.83 ± 4.75^b^	47.81 ± 8.92^b^
7.5 mg/kg albendazole	79.45 ± 5.03^c^	99.58 ± 3.84^c^	98.83 ± 0.80^c^	99.55 ± 0.91^c^

*Note:* For the same column, values carrying the same superscript letter are not significantly different at *p* ≥ 0.05 (*t*‐test).

### 3.3. Evaluation of Anthelmintic Efficacy of EB Oral Solution Against GIN Using mPCR

The eggs isolated from feces showed the presence of seven species of nematodes: *Haemonchus contortus*, *Trichostrongylus vitrinus*, *Trichostrongylus axei*, *Trichostrongylus colubriformis*, *Nematodirus filicollis*, *Oesophagostomum* spp., and *Ostertagia ostertagi*.

At Day 7 after treatment, mPCR showed the amplification of one species in 270 mg/kg EB oral solution group (*Haemonchus contortus*), two to three species in 90 mg/kg EB oral solution group (*Haemonchus contortus*, *Oesophagostomum* spp., and/or *Nematodirus filicollis*), and two species in positive control group (*Haemonchus contortus* and *Oesophagostomum* spp.) (Table 3).

**Table 3:** Efficacy of EB result from DNA isolated from fecal egg samples of goats at Day 7.

**Nematodes species**	**270 mg/kg**	**90 mg/kg**	**Positive control**
*Haemonchus contortus*	+	+	+
*Trichostrongylus vitrinus*	—	—	—
*Trichostrongylus axei*	—	—	—
*Trichostrongylus colubriformis*	—	—	—
*Oesophagostomum* spp.	—	+	+
*Nematodirus filicollis*	—	+	—
*Ostertagia ostertagi*	—	—	—

On 28th day, mPCR showed the amplification of one species in 270 mg/kg EB oral solution (*Haemonchus contortus*); two species in 90 mg/kg EB oral solution (*Haemonchus contortus* and *Oesophagostomum* spp.); and three species in positive control (*Haemonchus contortus*, *Trichostrongylus axei*, *and Oesophagostomum* spp.) (Table 4).

**Table 4:** Efficacy of EB result from DNA isolated from fecal egg samples of goats at Day 28.

**Nematodes species**	**270 mg/kg**	**90 mg/kg**	**Positive control**
*Haemonchus contortus*	+	+	+
*Trichostrongylus vitrinus*	−	−	−
*Trichostrongylus axei*	−	−	+
*Trichostrongylus colubriformis*	−	−	−
*Oesophagostomum spp*	−	+	+
*Nematodirus filicollis*	−	−	−
*Ostertagia ostertagi*	−	−	−

### 3.4. Toxicity Assessment of Oral Solution of EB

#### 3.4.1. Clinical Observations

During the 28 days of observation period, neither deaths nor clinical symptoms were reported in any of the treatment groups (90 mg/kg, 270 mg/kg of EB oral solution, and 7.5 mg/kg albendazole as positive control) (Table 5). The animal′s rectal temperature ranged between 37.85°C and 39.00°C, which was within normal range for small East African goats. Following the three (3) treatment groups, goats did not have significantly different body temperatures (*p* > 0.05). Before treatment, the goats in the albendazole and 270 and 90 mg/kg of EB oral solution groups weighed, respectively, 25.45 ± 1.2, 26.24 ± 2.4, and 26.76 ± 4.5 kg. After 3 weeks since treatment, the weight of those in the albendazole and 270 and 90 mg/kg oral solution groups was 25.75 ± 0.6, 27.73 ± 2.1, and 27.5 ± 2.9 kg, respectively, while in the untreated group, the body weight decreased from 16.50 ± 1.00 to 16.13 ± 0.63 kg. At 28 days after treatment with EB, the average body weight had increased by between 0.75 and 1.5 kg, and this increase was statistically significant (*p* < 0.05) compared to Day 0.

**Table 5:** Acute toxicity of EB oral solution administered in three doses to goats.

**Clinical signs**	**Animal groups**		
	**90 mg/kg EB**	**270 mg/kg EB**	**Positive control**
Change of kin	Normal	Normal	Normal
Mucous membrane (observed for abnormal color, presence of blood, sloughing off, etc.)	Normal	Normal	Normal
Liquid secretion from eyes	Absent	Absent	Absent
Respiratory system (rapid breathing and difficulty breathing)	Normal	Normal	Normal
Excessive salivation	Absent	Absent	Absent
Urination	Normal	Normal	Normal
Diarrhea	Absent	Absent	Absent
Sleep in the animal after drug administration	Absent	Absent	Absent
Lethargy (inactivity of the animal when compared to the control group)	Absent	Absent	Absent
Food taking	Normal	Normal	Normal
Water taking	Normal	Normal	Normal
Mortality associated with the drug administered	Nil	Nil	Nil

*Note:* Positive control = 7.5 mg/kg albendazole.

#### 3.4.2. Effects of Treatments on PCV and Biochemical Parameters

Prior to treatment, PCV levels in goats treated with albendazole and 270 and 90 mg/kg EB oral solution were, respectively, 30.73%, 29.55%, and 30.44%. During the medical treatment, the PCV levels varied between 28.58% and 30.25% for the 270 mg/kg EB oral solution group, 27.25% and 31.50% for the 90 mg/kg EB oral solution group, and 28.84% and 30.73% for the positive control group (Table 6). There were no statistically significant differences between the treatment groups (*p* > 0.05).

**Table 6:** Effect of EB oral solution on PCV in goats.

**Group**	**Day 0 (%)**	**Day 7 (%)**	**Day 14 (%)**	**Day 21 (%)**	**Day 28 (%)**
270 mg/kg EB	29.55 ± 3.27	30.25 ± 3.59	29.25 ± 2.21	28.58 ± 3.40	28.95 ± 1.79
90 mg/kg EB	30.44 ± 3.63	31.00 ± 4.16	30.00 ± 3.16	27.25 ± 2.06	31.50 ± 3.31
Positive control	30.73 ± 2.27	30.15 ± 4.26	30.01 ± 3.81	29.49 ± 2.67	28.84 ± 3.10
Negative control	29.50 ± 1.47	29.50 ± 4.12	29.75 ± 3.77	25.25 ± 2.21	22.75 ± 2.21

*Note:* Positive control = 7.5 mg/kg albendazole.

**Figure 1:**
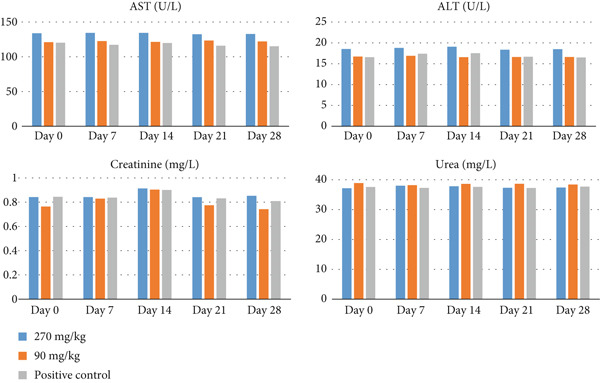
AST, ALT, creatinine, and urea levels of goats treated with EB oral solution and albendazole.

The average biochemical levels of AST, ALT, creatinine, and urea for each dosed group were within normal ranges during the monitoring period. The AST levels were 133.08 ± 0.56, 122.02 ± 0.92, and 117.62 ± 2.30 U/L for 270 and 90 mg/kg EB and albendazole, respectively, when the negative control was 105.03 ± 22.34 U/L. While ALT levels were 18.6 ± 0.27, 16.69 ± 0.13, 16.94 ± 0.48, and 16.31 ± 0.27 U/L for 270 and 90 mg/kg EB, albendazole, and untreated goats, respectively, creatinine levels were 0.85 ± 0.03 mg/L for the 270 mg/kg group and 0.76 ± 0.04 mg/L for the 90 mg/kg group, while urea levels’ means were 37.49 ± 0.4 for the 270 mg/kg group and 38.42 ± 0.27 for the 90 mg/kg (Figure 1). There were no significant changes (*p* > 0.05) in levels of AST, ALT, creatinine, and urea across treatment groups and days.

#### 3.4.3. Findings From Autopsies

At postmortem examination, there were no visible abnormalities in the animal′s organs and tissues. The organs in the EB oral solution treatment group (270 and 90 mg/kg) were healthy and comparable to the positive control group (albendazole). Microscopic examination of the liver and kidney from EB oral solution showed normal hepatic (hepatocytes and hepatic sinusoids) and renal (normal appearance of glomerulus) structures, respectively. Additionally, the heart and spleen were also in good condition (Figure 2). No microscopic abnormalities were detected in the organs of either the EB oral solution treatment or the positive control groups.

Figure 2Histology observation of kidney, heart, liver, and spleen of goats treated by EB. (a) Control group and (b) treatment group, showing no changes (microscope × 100 magnification).

**Figure:**
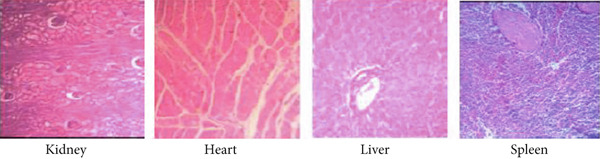
(a)

**Figure:**
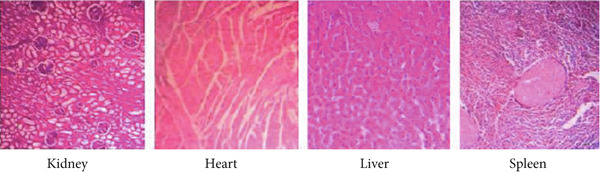
(b)

## 4. Discussion

The oral solution of EB was made in response to concerns about making the drug more useful against intestinal parasites. This current study demonstrated that the oral solution of EB made had a higher proteolytic activity and a lower pH than crude EB reported previously [19, 21]. The higher activity of the EB oral solution might be attributed to the lower pH, which aided in the faster release of bromelain from chitosan nanocarriers during the casein protease test. In addition, the presence of xanthan gum as an excipient in the formulation of the current medication would have impacted the degradability [36]. Al Remawi et al. demonstrated that the dissolution was similar to the commercial product when using chitosan and xanthan gum as excipients to control the release of ambroxol [36].

Similar findings with regard to the reduction of GIN egg excretion were reported previously [1, 20, 37, 38]. Wasso et al. showed a 68% efficacy on *Haemonchus contortus*, with 30 mg/kg for 14 days when the same EB was administered with water [20]. While Cormanes et al. found that pineapple (*Ananas comosus)* fruit peeling juice was effective against chicken gastrointestinal helminths on the 7th day posttreatment after administration of 1008 mg/kg body weight [38]. When sheep were given powdered leaves and plain bromelain, Hordegen et al. [37] and Domingues et al. [1] found that their anthelmintic potency increased.

The significant decrease in egg production may be attributable to adult cuticle damage. Bromelain is known for its distinctive phytochemicals, which have the potential to inflict damage on the cuticle of nematodes [38, 39]. Transverse wrinkles were formed on the worm cuticle, which was followed by the shrinkage and blistering of cretes, as documented by [39].

Thus, the present study corroborates the previous findings observed with cysteine proteinase from other plants [39–41]. These experiments reveal that the decrease in egg excretion observed *in vivo* may be due to a reduction in female worm prolificity or adult worm mortality. Stepek et al. [39] discovered that papaya latex significantly reduced the worm burden and egg production in rodent GIN (*Trichuris muris* and *Heligmosomoides polygyrus*). On sheep, Pascal et al. [40] reported that *Newbouldia laevis* (Bignoniaceae) was 55% effective against *Haemonchus contortus* and 19% effective against *Trichostrongylus colubriformis* when administered. Hounzangbe‐Adote et al. [42] reported that after 10–15 days of treatment, papaya (*Carica papaya*) seed powder was 80% effective against sheep strongylus.

In the current study, mPCR showed that EB oral solution was effective on *T. vitrinus*, *T. axei*, *T. colubriformis*, *Oesophagostomum* spp., *N. filicollis*, and *O. ostertagi* species. However, *H. contortus* DNA continued to be amplified up to Day 7 posttreatment. The amplification of *H. contortus* DNA could be due to high burdens manifested by this worm [43, 44]. *H. contortus* is also located in the abomasum whose low pH impedes the activity of bromelain. The activity of bromelain may also be ineffective against the parasite in the abomasal mucosa where it is not accessible to bromelain [39]. The fact that no DNA of other GIN was observed at Day 7 posttreatment is a good indicator that EB was able to pass to the small intestine in good concentrations leading to the elimination of the other intestinal nematodes located in this section of the gut. Indeed, the study by Wasso et al. [20] showed that a good concentration of EB is sustained throughout the gut. The effectiveness of using PCR as a tool for the determination of the efficacy of drugs against GIN species has been reported by previous studies [45–47]. Mondragón‐Ancelmo et al. [46], in their study, identified through PCR that *Cooperia* spp. and *Trichostrongylus colubriformis* from sheep were resistant to Albendazole and Ivermectin. Roeber et al. [45] demonstrated the sensitivity (98%) and specificity (100%) of PCR as a diagnostic tool to assess the composition of strongylid nematode populations in sheep with naturally acquired infections.

The toxic effect of EB oral solution on goats was also assessed. In this study, there were no clinical signs or mortalities, according to a recent study that evaluated the acute toxicity [20]. Conversely, the treated animals experienced minor weight gains, which may indicate that the drug improved the goats′ growth performance. Cormanes et al. [38] observed a comparable phenomenon following the administration of pineapple (*Ananas comosus*) fruit paring liquid to poultry. They reported a substantial increase in weight gain in comparison to the placebo group. However, the animal′s body would be able to more effectively utilize the nutrients in the diet as a result of the nematode elimination, which would result in weight gain.

In the current study, the biochemical parameters were within the normal range and were comparable to the results reported by [20]. Wasso et al. [20] treated goats with EB at a dose of 30 mg/kg for 14 days and at a single dose of 90 and 270 mg/kg. The absence of macroscopic and microscopic alterations in the examined organs also confirmed this [48, 49]. After administering bromelain to canines at a dosage of 750 mg/kg per day for 6 months, Pavan et al. [49] did not observe any toxic effects. These findings indicate that the medication is generally safe for use in a variety of animals.

## 5. Conclusion

Chitosan EB was shown to be effective in fighting goats′ GIN, with a substantial decrease in FECs. The study found that an oral solution made with excipient such as xanthan gum increased EB effectiveness. Additionally, the oral solution of EB did not exhibit any adverse effects on goats at a concentration of 270 mg/kg. Consequently, this bromelain formulation has the potential to contribute to the regulation of GIN. It is imperative to conduct additional research in order to optimize the formulation of the oral solution of EB and to adapt the dosage regimens to enhance the efficacy of GIN in small ruminants.

## Conflicts of Interest

The authors declare no conflicts of interest.

## Funding

This work was supported by the Pan‐African University of Institute of Basic Science, Technology, and Innovation (PAUSTI).

## Data Availability

The data that support the findings of this study are available from the corresponding author upon reasonable request.
